# Effects of aged black garlic (ABG10+^®^) on lipoprotein subclass composition in Grade I hypertension: a randomized, triple-blind controlled trial with sex-stratified sequential allocation

**DOI:** 10.3389/fnut.2026.1869617

**Published:** 2026-07-27

**Authors:** José C. E. Serrano, Eva Castro-Boqué, Alicia García-Carrasco, María Inés Morán-Valero, Marina Díez Municio, Marcelino Bermúdez-López, José Manuel Valdivielso, Alberto E. Espinel, Manuel Portero-Otín

**Affiliations:** 1 NUTREN-Nutrigenomics, Department of Experimental Medicine, University of Lleida, and Biomedical Research Institute of Lleida – Dr. Pifarré Foundation, IRBLleida, Lleida, Spain; 2 Vascular and Renal Translational Research Group, Biomedical Research Institute of Lleida – Dr. Pifarré Foundation, IRBLleida, Lleida, Spain; 3 Pharmactive Biotech Products S.L.U., Madrid, Spain

**Keywords:** aged black garlic, lipid profile, lipoproteins, S-allyl-cysteine, triacylglycerides

## Abstract

**Background:**

Aged black garlic (ABG) is rich in organosulfur compounds such as S-allyl-cysteine (SAC) and may influence lipid metabolism, although evidence from controlled trials remains limited. The aim of this study was to evaluate the impact of a low-dose SAC-optimized ABG on blood lipid profiles in Grade I hypertensive individuals.

**Methods:**

A randomized, triple-blind, placebo-controlled trial with systematic sex-stratified sequential allocation was conducted with 75 Grade I hypertensive participants receiving either 250 mg of ABG or a placebo daily for 12 weeks. Plasma lipoproteins and subclass composition were quantified by NMR spectroscopy, and metabolic clusters were explored using k-means and PLS-DA.

**Results:**

ABG supplementation did not produce statistically significant changes in any conventional lipid marker (total cholesterol, LDL-C, HDL-C, or triglycerides). In secondary exploratory analyses of NMR-derived lipoprotein subclasses, nominally significant differences were observed: ABG was associated with a reduction in the total number of particles and HDL particles. Detailed analysis of XXL-VLDL particles showed a decrease in the percentage of free and esterified cholesterol alongside an increase in triglyceride percentage. Large HDL particles showed compositional remodeling characterized by an increase in phospholipid content and a decrease in triglyceride percentage. In exploratory cluster analyses, participants with a more adverse baseline metabolic profile showed a nominally significant reduction in total triglycerides and VLDL-lipid content after ABG intake.

**Conclusion:**

Low-dose ABG supplementation did not significantly modify conventional lipid markers in Grade I hypertensive individuals. However, secondary exploratory NMR-based analyses suggest that ABG may induce compositional remodeling of lipoprotein subclasses—particularly reducing the cholesterol load in large VLDL particles and enhancing HDL phospholipid content—especially in individuals with a more adverse baseline metabolic profile. These findings are hypothesis-generating and require confirmation in adequately powered trials with pre-specified NMR endpoints.

**Clinical Trial Registration:**

identifier: NCT04915053.

## 1 Introduction

Garlic (Allium sativum) has been used for centuries in traditional medicine for its health benefits. Ancient civilizations like the Egyptians, Greeks, Romans and in traditional Chinese and Ayurvedic medicine considered it as a formidable prophylactic and therapeutic medicinal agent (1). Because of the widespread effects of garlic in maintaining good health, it has attracted particular attention of modern medicine. The effect of garlic in dyslipidemia was suggested several years ago (2). A recent meta-analysis has found at least 142 articles related to garlic and dyslipidemia (3) in which it is suggested that a significant reduction in Total-cholesterol (TC), Low-density-lipoprotein (LDL) cholesterol and triacylglycerides (TG) blood levels. This is due to its composition of bioactive compounds mainly phenolic and organosulfur compounds (4), that has been proposed to reduce hepatic lipogenesis and cholesterolegenic enzymes (5); enhanced excretion of acidic and neutral steroid into bile salts (6) and suppressed LDL oxidation (7).

However, one of the main problems with these findings is that its translation to clinical practice could be damped since the required doses of garlic to observe the desired effects are relatively high and undesirable symptoms may be observed. Aged Black Garlic (ABG) has gained popularity over regular garlic as a therapeutic treatment due to its enhanced bioavailability, milder taste, and increased antioxidant properties. The fermentation process that transforms fresh garlic into ABG reduces its pungent odor and harsh flavor, making it more appealing for consumption (8). Additionally, this process increases the concentration of bioactive compounds such as S-allyl-cysteine (SAC), which has been linked to improved cardiovascular health, anti-inflammatory effects, and stronger antioxidant activity (9). Unlike raw garlic, ABG is also easier to digest, reducing the risk of gastrointestinal discomfort. These factors have contributed to its growing use in alternative medicine and functional foods (10).

Several scientific studies have compared the effects of ABG and raw garlic on blood lipid and cholesterol profiles. Research suggests that both types of garlic can reduce TC, LDL cholesterol, and TG, while increasing high-density-lipoprotein (HDL) cholesterol (11). However, ABG appears to have a more potent effect due to its higher concentration of bioactive compounds such as SAC and polyphenols (12).

Nevertheless, while traditional lipid profiles focus on TC, LDL-cholesterol, HDL-cholesterol, and TG; studying lipoprotein subclasses provides a more detailed and accurate assessment of cardiovascular risk. LDL is not a single entity; small, dense LDL (sdLDL) particles are more atherogenic than larger, more buoyant LDL particles. Small LDL particles penetrate the arterial wall more easily and are more prone to oxidation, increasing the risk of plaque formation (13). Studies show that individuals with high sdLDL levels have a greater risk of CVD even if their overall LDL cholesterol levels appear normal (14). Similarly, not all HDL particles are equally protective. Large HDL particles are more effective in reverse cholesterol transport, whereas small HDL particles may be dysfunctional (15). In the same context, low HDL particle concentration and dysfunctional HDL composition have been associated with increased cardiovascular risk, independent of total HDL-cholesterol levels (16). Last, smaller VLDL particles are more readily converted into LDL, particularly sdLDL, which is highly atherogenic. Elevated levels of sdLDL are associated with endothelial dysfunction, increased oxidative stress, and enhanced arterial plaque formation, all of which contribute to the progression of atherosclerosis and cardiovascular events (17). Therefore, analyzing blood lipoprotein particle size provides valuable insight into cardiovascular risk beyond traditional lipid measurements.

Hypertension represents a key clinical context in which examining lipid and lipoprotein profiles is particularly relevant. Hypertensive individuals frequently exhibit subtle but clinically meaningful abnormalities in lipid metabolism, even in the absence of overt dyslipidemia (18). Studies show that hypertension commonly coexists with increased triglyceride-rich VLDL particles, higher concentrations of small, dense LDL, and reduced levels of large HDL particles—patterns that exacerbate endothelial dysfunction, inflammation, and arterial stiffness (19–21). The coexistence of hypertension and subclinical dyslipoproteinemia amplifies atherosclerotic processes through synergistic metabolic and vascular mechanisms. Therefore, assessing detailed lipid and lipoprotein traits in hypertensive subjects provides important insight into early cardiometabolic derangements and offers a relevant target population for evaluating functional foods with potential lipid-modulating effects. Even modest improvements in lipoprotein dynamics may yield clinically meaningful benefits in this group.

In this context, the present study had two pre-specified objectives. The primary objective was to evaluate whether daily supplementation with a low-dose, SAC-optimized ABG extract for 12 weeks modifies conventional blood lipid markers (total cholesterol, LDL-cholesterol, HDL-cholesterol, and triglycerides) in individuals with Grade I hypertension receiving stable antihypertensive therapy. The secondary objective was to characterize the effect of ABG on NMR-derived lipoprotein subclass composition and particle number, which provide mechanistic resolution beyond standard lipid panels. Given the inherently greater biological variability and smaller expected effect sizes of these advanced parameters, and consistent with the power calculation described in Section 2.4, NMR-derived findings are designated as exploratory secondary outcomes and should be interpreted as hypothesis-generating. LP-IR and other metabolically related biomarkers were included as complementary variables to provide integrated metabolic context. Additionally, tertiary analyses aimed to determine whether specific metabolic phenotypes exhibit differential responsiveness to ABG supplementation, acknowledging the heterogeneity typically present in hypertensive populations.

## 2 Methods

### 2.1 Experimental design

The study was designed as a triple-blind, placebo-controlled, parallel trial with systematic sex-stratified sequential allocation; to assess whether an optimized aged black garlic (ABG) extract could improve the blood lipid profile in subjects with Grade I hypertension. Volunteers were sequentially allocated into two groups—Group ABG and Group Placebo—and treated for 12 weeks with the active ingredient or placebo, respectively. The treatment duration was determined based on previous studies reported in various meta-analyses on garlic blood lipoproteins (3, 22, 23).

**Figure 1:**
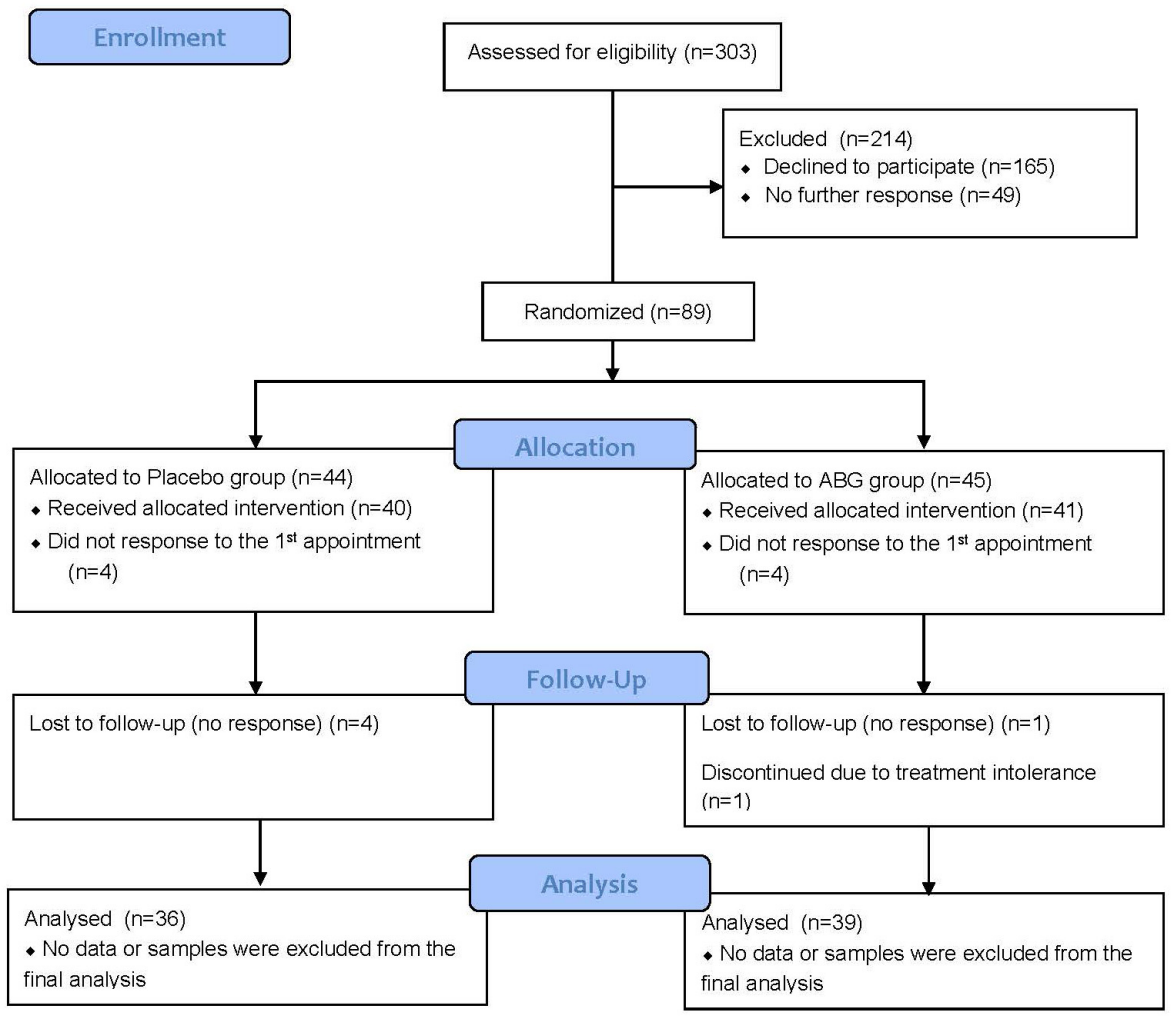
Flow chart of study design and volunteers' distribution and dropouts in each phase.

Volunteers were assigned to each group using a systematic, sex-stratified sequential allocation procedure after agreeing to participate in the study; participants were identified solely by a sequential numeric code without access to prior personal identifying data. A formal computer-generated randomization sequence with pre-specified block sizes and a formal allocation concealment mechanism (e.g., sealed opaque envelopes or independent third-party allocation) were not implemented; this is acknowledged as a methodological limitation in Section 5. The personnel who enrolled and those who assigned participants to the interview had no access to the allocation code thereafter, and treatment allocation remained concealed from participants, outcome assessors, and the statistician throughout the study (triple-blind design). Statistical analyses were conducted on a per-protocol basis, including only participants who completed the 12-week intervention with complete baseline and post-intervention data (*n* = 75 of 89 randomized; 15.7% overall attrition); an intention-to-treat analysis was not performed. Attrition did not differ significantly between the ABG (6/45, 13.3%) and placebo (8/44, 18.2%) arms (Fisher's exact test, *p* = 0.57), as detailed in the CONSORT flow diagram (Figure 1). The study was conducted in accordance with the ethical principles outlined in the latest versions of the Declaration of Helsinki and Tokyo for human research, as well as Regulation (EU) 2016/679 of the European Parliament and Council of April 27, 2016, on Data Protection (GDPR); Law 14/2007 on biomedical research; and Organic Law 3/2018 on the Protection of Personal Data and Guarantee of Digital Rights. Participants provided informed consent before joining the study. The study protocol was approved by the Hospital Universitari Arnau de Vilanova (CEIC-2435), Lleida, Spain, and the trial is registered at ClinicalTrials.gov under the identifier NCT04915053. This trial was designed and reported in accordance with the CONSORT 2010 Statement for randomized controlled trials. No changes to the methods were introduced after the trial commenced.

### 2.2 Intervention

The intervention consisted of the daily intake of one tablet upon waking and before breakfast. The ABG group received a tablet of optimized aged black garlic extract (Abg10+^®^/Garlace^®^), standardized in Agedgarlides^®^ (a totum of the main organosulfur compounds found in aged garlic, responsible for the mechanism of action and the scientifically proven bioactivity) and manufactured by Pharmactive Biotech Products, S.L.U. (Madrid, Spain) through ActiveNature^®^ Technology. The extract was derived from garlic grown in the Las Pedroñeras region (Castilla-La Mancha, Spain), and the tablets were produced by Instant Procès (La Roca del Vallès, Spain). Each ABG tablet contained 250 mg of ABG extract, equivalent to 0.25 mg of SAC per tablet, along with 300 mg of excipients. Detailed composition is described in Table 1. The placebo group received a tablet in which the ABG extract was replaced with maltodextrin. Both tablets were similar in appearance and odor and were coded by the manufacturer. To ensure blinding, none of the researchers involved in the study knew to which group each tablet had been assigned. Participants were instructed to maintain their dietary habits, physical activity, and medical treatments. Adverse events were asked systematically in each interview. No interim analysis was performed. At the end of the experimental phase, any changes in lifestyle and pharmacological treatment were assessed, with no volunteers reporting modifications. Volunteer recruitment and the start of the trial began in September 2021 and concluded in April 2022.

**Table 1:** Detailed composition of tablets employed in the study.

Ingredient	Placebo (mg per tablet)	ABG extract (mg per tablet)
ABG extract	-	250
Maltodextrin	250	-
Microcrystalline cellulose	90	90
Dicalcic phosphate	157	157
Sodium croscarmellose	10	10
Magnesium stearate	7	7
Sodium alginate	3.06	3.06
Stearic acid	0.03	0.03
Oleic acid	1.54	1.54
Medium-chain triglycerides	2.80	2.80
Ethylcellulose	13.17	13.17
Hydroxypropyl methylcellulose	4.86	4.86
Hydroxypropyl cellulose	4.86	4.86
Talc	2.88	2.88
Titanium oxide	1.80	1.80
Garlic flavor	1.00	1.00

### 2.3 Study population

Volunteers were recruited in collaboration with the Atherothrombotic Disease Detection and Treatment Unit (UDETMA) at the Hospital Universitari Arnau de Vilanova (Lleida, Spain) from a database of patients diagnosed with Grade I hypertension. The inclusion criteria encompassed men and women over 18 years old with hypertension under treatment with one or more antihypertensive drugs, excluding Angiotensin-Converting Enzyme (ACE) inhibitors and angiotensin receptor blockers (ARBs). This exclusion was adopted as a precautionary safety measure: although garlic-derived compounds do not negatively interfere with the antihypertensive mechanism of renin-angiotensin system (RAS) inhibitors, the available clinical evidence on this specific combination is limited and the magnitude of any additive hypotensive interaction is variable across studies. In the absence of robust data, a conservative criterion was adopted to minimize the risk of symptomatic hypotension in participants already receiving antihypertensive pharmacotherapy. Exclusion criteria included morbid obesity (body mass index >35 kg/m^2^), serum LDL-cholesterol levels below 115 mg/dL (threshold for low or moderate SCORE risk of cardiovascular events), fasting serum glucose levels above 126 mg/dL (cut-off for diabetes diagnosis), anemia (hemoglobin < 13 g/dL in men and < 12 g/dL in women), chronic gastrointestinal disease, pregnancy or intention to become pregnant, lactation, participation or planned participation in a clinical trial or nutritional intervention study within the 30 days prior to enrolment, and allergy to garlic.

### 2.4 Sample size determination

The sample size was estimated based on available evidence regarding the variability of traditional lipid parameters and the expected lipid-lowering effects of garlic-derived preparations. Within-person variability for total cholesterol has been reported to be low (SD = 0.12 mmol/L) (24), and meta-analytic data suggest that garlic supplementation may reduce total cholesterol by approximately 0.37 mmol/L (23). Under these assumptions, a sample size of approximately 36 participants per group provides very high statistical power to detect changes of this magnitude using a two-sided α = 0.05. In addition to traditional lipid measures, the present study incorporated NMR-derived lipoprotein subclasses and LP-IR as complementary metabolic variables. These advanced parameters typically exhibit greater biological variability and smaller expected effect sizes than routine lipids. Therefore, although the study was adequately powered for detecting moderate changes in conventional lipid markers, it was not specifically powered to detect small or subtle modifications in lipoprotein particle composition. Findings related to these exploratory outcomes should thus be interpreted as hypothesis-generating. Accordingly, conventional lipid markers (total cholesterol, LDL-cholesterol, HDL-cholesterol, and triglycerides) constitute the primary outcomes of this study, while NMR-derived lipoprotein subclass parameters are designated as secondary exploratory outcomes.

### 2.5 Outcomes

#### 2.5.1 Blood lipid profile

Lipid profiles were investigated by the Nightingale Health's NMR spectroscopy method (25–27). Following the blood draw, blood samples were stored at −80 °C prior to shipment to the Metabolomics Core Facility. Two hundred fifty biomarkers were determined which included routine lipids, apolipoproteins, amino acids, fatty acids, lipoprotein particles, inflammation, and glycolysis-related metabolites.

#### 2.5.2 Lipoprotein insulin resistance index (LP-IR)

LP-IR was calculated from NMR-derived lipoprotein parameters (28). The index integrates six lipoprotein subclass features associated with insulin-resistant dyslipidemia: large VLDL particle concentration, small LDL particle concentration, large HDL particle concentration, and the mean particle sizes of VLDL, LDL, and HDL. For each participant, LP-IR was computed on the 0–100 scale, where higher values indicate a more insulin-resistant lipoprotein pattern.

#### 2.5.3 Anthropometric measurements

Anthropometric measurements were obtained following standardized procedures. Body weight was measured to the nearest 0.1 kg using a calibrated scale, with participants wearing light clothing and no shoes. Height was measured to the nearest 0.1 cm using a wall-mounted stadiometer, ensuring an upright posture with heels, buttocks, and upper back aligned. Body mass index (BMI) was calculated as weight divided by height squared (kg/m^2^). Waist and hip circumferences were assessed using a non-elastic tape placed horizontally at standardized anatomical landmarks. Waist circumference was measured midway between the lowest rib and the iliac crest after gentle exhalation, and hip circumference at the level of the greatest gluteal protrusion. Two measurements were taken for each parameter, and a third measurement was performed when the first two differed by more than 0.5 cm. The waist-to-hip ratio was calculated as waist circumference divided by hip circumference.

#### 2.5.4 Metabolic syndrome classification

Metabolic syndrome classification was done according to the Adult Treatment Panel III (ATP III) of the National Cholesterol Education Program (NCEP) guidelines (29). Metabolic Syndrome was classified on the presence of at least three out of five criteria that include: abdominal obesity (waist circumference >102 cm in men and >88 cm in women), elevated triglycerides (≥150 mg/dL or drug treatment for high triglycerides), low HDL cholesterol (< 40 mg/dL in men and < 50 mg/dL in women or drug treatment for low HDL), high blood pressure (≥130/85 mmHg or use of antihypertensive medication), and elevated fasting glucose (≥100 mg/dL or current treatment for hyperglycemia).

#### 2.5.5 Endothelial function

Endothelial function was evaluated using the EndoPAT™ parameters, including the reactive hyperemia index (RHI) and the augmentation index (AI_75_), both assessed at baseline and after 12 weeks of intervention. Analyses were conducted in a room maintained at a thermoneutral temperature of 24 °C. Volunteers were instructed to remove any restrictive clothing, watches, or rings that could hinder blood flow to the arms and fingers. The blood pressure cuff was then snugly placed on the upper arm. Participants lay comfortably in the study room, relaxing for at least 15 min before the test began. The measurement protocol comprised 1 min of initial reading to verify proper sensor placement and signal quality, followed by 5 min under basal conditions, 5 min of occlusion, and 5 min of recording post-occlusion. Occlusion was achieved by inflating the cuff to a supra-systolic level, at least 60 mmHg above the systolic blood pressure and not below 200 mmHg. Blood-flow cessation was confirmed by the total absence of signal from the occluded hand. If any movement was detected, cuff pressure was increased by an additional 50 mmHg, up to a maximum of 300 mmHg. The EndoPAT™ 2000 software automatically calculated RHI and AI_75_ values. Occlusion parameters were reviewed for all measurements, with manual adjustments made as needed.

#### 2.5.6 Blood pressure

Blood pressure measurements were obtained using the same oscillometric automatic sphygmomanometers (Medisana BU 512, Medisana GmbH, Neuss, Germany), with a maximum static pressure error tolerance of ±3 mmHg. Volunteers remained seated comfortably in a quiet environment for 10 min before the measurements. Blood pressure values were determined by averaging three consecutive readings of systolic and diastolic blood pressure, taken at 3-min intervals while participants were seated with their feet flat on the floor and arms supported at heart level.

### 2.6 Statistical analysis

Primary and secondary parameters were measured at baseline and after 12 weeks of intervention. All the data was included in the statistical analysis, and no missing data was observed. Data are presented as mean ± 95% confidence interval of the mean. Two-way ANOVA and multiple comparisons, used to analyze the treatment effect, were performed using Sidak's test. A statistical analysis of initial differences between Groups ABG and placebo was performed by a two-tailed unpaired Student's *t*-test. A Chi-square test was performed for categorical variables.

A multivariable linear regression model was used to evaluate the association between the change in blood pressure and lipoprotein subclasses. The model included an interaction term between change in lipoproteins and study group (Placebo vs. ABG) to assess whether the association differed between groups. The analysis was adjusted for age, sex, body mass index (BMI), and previous lipid-lowering medication. A two-sided *p-value* < 0.05 was considered statistically significant.

To identify distinct metabolic patterns within the lipoprotein profile data, k-means clustering was performed using the MetaboAnalyst 6.0 platform (https://www.metaboanalyst.ca/). The input variables comprised the full panel of 255 baseline NMR-derived lipoprotein parameters, including concentration and particle size of VLDL, LDL, and HDL subclasses (XXL-, XL-, L-, M-, S-, and XS-sized fractions), their lipid composition (total lipids, triglycerides, phospholipids, free cholesterol, and esterified cholesterol as absolute concentrations and percentages), total lipoprotein particle number, and the lipoprotein insulin resistance index (LP-IR). All variables were standardized (z-score) prior to clustering. The number of clusters was set a priori to *k* = 3, based on a pre-specified clinical hypothesis: given the well-established heterogeneity of metabolic syndrome (MetS) severity, participants were expected to fall into three metabolically distinct subgroups—(1) a cluster with a relatively favorable metabolic profile and few or no MetS features; (2) an intermediate cluster with moderate metabolic alterations; and (3) a cluster with pronounced MetS characteristics, including elevated triglycerides, dyslipidemia, and insulin resistance. This tripartite classification is consistent with current frameworks of cardiometabolic risk stratification. To provide statistical support for this choice, the elbow method (within-cluster sum of squares) and mean silhouette score were evaluated retrospectively across *k* = 2 to *k* = 10 (Supplementary Figure S2). The elbow plot indicated an inflection at *k* = 3, after which the reduction in within-cluster variance became progressively marginal. The silhouette score at *k* = 3 (0.222) was consistent with the values observed across the evaluated range. Although *k* = 2 yielded a marginally higher silhouette score (0.240), this two-cluster solution would collapse the intermediate and high-MetS subgroups into a single partition, precluding the clinically meaningful stratification central to our hypothesis. Further, to identify the distinguishing features of each cluster, Partial Least Squares Discriminant Analysis (PLS-DA) using the MetaboAnalyst platform was applied. To assess the statistical differences of each variable across clusters, volcano plots within the MetaboAnalyst platform were performed. To assess treatment effects on the lipoprotein profile, NMR-derived variables were organized into 14 pre-specified biologically coherent families based on lipoprotein class and lipid type: lipoprotein particles (*n* = 18), apolipoproteins (*n* = 3), particle size (*n* = 3), total lipids (*n* = 4), total cholesterol (*n* = 6), cholesteryl esters (*n* = 4), free cholesterol (*n* = 4), phospholipids (*n* = 4), triacylglycerides (*n* = 4), other lipids (*n* = 5), VLDL compositional percentages (*n* = 30), HDL compositional percentages (*n* = 20), IDL compositional percentages (*n* = 5), and LDL compositional percentages (*n* = 15), totalling 125 lipoprotein-related variables. Non-lipoprotein NMR metabolites (amino acids, glycolytic metabolites, and other small molecules) were outside the scope of the primary research question and were not included in these analyses. For each family, a two-way ANOVA with Group (ABG vs. Placebo) and Time (baseline vs. post-intervention) as between- and within-subject factors was performed in GraphPad Prism v9; the primary statistical endpoint was the Group × Time interaction term, which assesses whether the change over time differs between treatment groups. This family-based analytical strategy constitutes a form of implicit multiplicity control, as each ANOVA is applied to a biologically coherent set of variables rather than to all measured parameters simultaneously, with a modest number of comparisons per family (range: 3–30; mean: ~9). Finally, in all cases, the cut-off to determine significant differences was set at a *p-value* below 0.05. Statistical analyses and graphs were achieved with GraphPad Prism v9 and MetaboAnalyst platform.

## 3 Results

### 3.1 Study design

A flow chart of the study design and volunteer included in each phase is depicted in Figure 1. A total of 303 patients who met the inclusion and exclusion criteria were contacted; of these, 89 expressed interests in participating in the study, 165 were not interested, and 49 did not answer the phone call. The 89 patients interested in participating in the study were randomly assigned to two groups: 44 volunteers in the Placebo Group and 45 volunteers in the ABG Group. All of them were scheduled for an initial appointment to provide study instructions. Of the 89 volunteers scheduled, 8 did not attend (4 from the Placebo group and 4 from the ABG group) for the first appointment, leaving 81 volunteers who started the experimental phase. At the end of the experimental phase, 4 volunteers from the placebo group and 1 volunteer from the ABG group were excluded due to loss to follow up. Last, 1 volunteer from the ABG group discontinued the study due to treatment intolerance. Finally, the data and sample analysis of a total of 36 and 39 volunteers from the Placebo and ABG groups, respectively, were analyzed and included in the study results. After that, no data or samples were excluded from the final analysis. The baseline characteristics of the volunteers are described in Table 2. No significant differences were observed in the initial lipid profile parameters between the Placebo and ABG groups.

**Table 2:** Volunteer's basal characteristics.

Variable	Group Placebo	Group ABG	*p-value*
Number of volunteers	36	39	
Sex (male:female)	20:16	20:19	0.8178
Body mass index (kg/m^2^)	31.9 ± 5.4	30.9 ± 4.9	0.4011
Waist/hip ratio			
Men	1.02 ± 0.06	1.00 ± 0.07	0.4792
Women	0.90 ± 0.06	0.93 ± 0.07	0.1627
Lipid lowering treatment (yes/no)	20/16	16/23	0.2513
Blood glucose (mg/dL)	108 ± 16	106 ± 12	0.5547
Total blood lipids (mmol/L)	9.95 ± 1.74	9.23 ± 1.88	0.0883
Blood triacylglycerides (mmol/L)	1.46 ± 0.66	1.30 ± 0.66	0.2796
Total blood cholesterol (mmol/L)	5.33 ± 1.00	4.94 ± 0.97	0.0927
HDL-Cholesterol (mmol/L)	1.44 ± 0.40	1.41 ± 0.36	0.7011
LDL-Cholesterol (mmol/L)	2.25 ± 0.52	2.07 ± 0.48	0.1175
VLDL-Cholesterol (mmol/L)	0.71 ± 0.26	0.62 ± 0.26	0.1525
LP-IR score	39.6 ± 4.4	39.9 ± 4.4	0.8014

Data is presented as mean ± standard deviation. Differences between groups were determined by two-tailed t-student for parametric variables or chi-squared for non-parametric variables. P-value below 0.05 were considered as statistical differences between groups.

### 3.2 Treatment adherence and reported adverse events

Treatment compliance was calculated by dividing the number of pills taken by the total number of treatment days. Some volunteers extended their treatment period due to scheduling adjustments for appointments. The average number of treatment days was 87 ± 5 in ABG group and 88 ± 5 in Placebo group. Both groups showed a mean compliance rate above 96%, with no significant difference between the groups (*p* = 0.4396). All data was retained for statistical analysis. Adverse events were reported by five volunteers and did not interfere with treatment adherence. Two participants in the ABG group and two in the placebo group experienced mild gastric discomfort, while one additional participant in the placebo group reported muscle cramps. No severe adverse events were recorded.

To evaluate the blinding process, participants were asked at their final appointment whether they believed they were in the active or placebo group. In response, 23% of volunteers in the ABG group and 45% in the Placebo group believed they were in the active group, confirming that blinding was effective. The researchers also remained unaware of group assignments until the completion of the statistical analysis.

### 3.3 Lipid profile

Table 3 shows the effect of ABG treatment on the lipid profile. After 12 weeks of treatment, no significant change is observed in any of the usually analyzed biomarkers in clinical practice, nor in the LP-IR score and TG/HDL-cholesterol ratios. Similarly, it was analyzed whether the response to treatment differs based on the sex of the volunteer and whether they receive medication to reduce cholesterol (Supplementary Tables S1 and S2). It was observed that men significantly modified their total cholesterol levels, without observing differences in terms of the treatment received. Regarding the effect of lipid-lowering treatment, 30 volunteers reported the use of statins (13 Atorvastatin, 16 Simvastatin, and 1 Pravastatin) and one volunteer reported the use of Ezetimibe. It was observed that lipid-lowering treatment does not have any influence on the response to treatment with ABG.

**Table 3:** Changes in lipoprotein subclasses related to metabolic syndrome.

Variable	Group placebo			Group ABG			*p-value* for interaction
	Pre-treatment	Post-treatment	*p-value*	Pre-treatment	Post-treatment	*p-value*	
LDL-size (nm)	23.8 ± 0.07	23.9 ± 0.09	>0.9999	23.9 ± 0.08	23.9 ± 0.08	>0.9999	0.5919
HDL-size (nm)	9.53 ± 0.18	9.53 ± 0.18	>0.9999	9.50 ± 0.19	9.50 ±0.19	>0.9999	0.7650
VLDL-size (nm)	38.9 ± 1.3	38.8 ± 1.2	0.8240	38.6 ± 1.2	38.6 ± 1.2	>0.9999	0.7042
S-LDL-p (nM)	199.1 ± 45.3	199.6 ± 42.7	0.8651	180.9 ± 40.7	184.5 ± 4.12	0.3015	0.5292
L-HDL-p (mM)	1.27 ± 0.85	1.30 ± 0.89	0.4595	1.16 ± 0.74	1.18 ± 0.75	0.4003	0.9246
L-VLDL-p (nM)	12.7 ± 7.5	12.3 ± 6.2	0.7067	11.0 ± 7.5	12.7 ± 7.5	0.1175	0.1658
Triacylglycerides (mM)	1.46 ± 0.65	1.42 ± 0.53	0.4600	1.30 ± 0.67	1.31 ± 0.71	0.8345	0.3448
HDL-cholesterol (mM)	1.44 ± 0.40	1.46 ± 0.43	0.5203	1.40 ± 0.36	1.43 ± 0.38	0.2228	0.6806
TG/HDL-c ratio	1.12 ± 0.67	1.06 ± 0.52	0.3330	1.04 ± 0.75	1.05 ± 0.86	0.8502	0.7761
Total cholesterol (mM)	4.93 ± 0.98	5.07 ± 1.00	0.2043	5.33 ± 1.00	5.37 ± 0.98	0.8243	0.4167
LP-IR score	39.6 ± 4.4	39.5 ± 4.2	0.7247	39.9 ± 4.4	39.7 ± 5.9	0.7351	0.9844

Data is presented as mean ± standard deviation. Differences between groups was determined by two-way ANOVA. Bonferroni's multiple comparison test was used to determine differences by time and group treatment. P-value below 0.05 were considered as statistical differences between groups.

As a secondary exploratory analysis, the detailed NMR-derived lipoprotein profile is shown in Supplementary Figure S1, where the 95% confidence intervals of the difference between the Placebo and ABG groups after 12 weeks of treatment are depicted. Similarly, in Table 4 differences and 95% confidence intervals of the differences of the features that presented a statistically significant change are described. These findings should be interpreted in the context of the exploratory nature of the NMR analyses. Nominally significant differences were observed: a decrease in the total number of particles (Total-P) (*p* = 0.0136), primarily in HDL particles (*p* = 0.0252) in the ABG treated group was observed. Differences were also present in the composition of XXL-VLDL, where the ABG group showed a higher percentage of triglycerides (*p* < 0.0001) and a lower percentage of free and esterified cholesterol (*p* < 0.0001 in both) in XXL-VLDL particles. In contrast, in L-HDL particles the ABG group had a lower percentage of triglycerides (*p* < 0.0036) and a higher percentage of phospholipids (*p* < 0.0277) compared to the placebo.

**Table 4:** Main lipoprotein particles difference between treatments.

Variable	Mean difference (Placebo – ABG+)	95% CI of the difference	*p-value*
Total particles	2.7 x 10^−4^	5.7 x 10^−5^ to 4.9 x 10^−4^	0.0136
HDL-particles	2.5 x 10^−4^	3.1 x 10^−5^ to 4.7 x 10^−4^	0.0252
XXL-VLDL-C%	13.8	9.6 to 18.1	< 0.0001
XXL-VLDL-CE%	13.1	8.8 to 17.3	< 0.0001
XXL-VLDL-TG%	−11.28	−15.5 to −7.1	< 0.0001
L-HDL-PL%	−1.98	−3.7 to −0.2	0.0277
L-HDL-TG%	2.63	0.9 to 4.4	0.0036

The data is presented as the main differences between Placebo and ABG+ treatment. Two way ANOVA was used to determine treatment effect. Bonferroni's multiple comparison test was used to determine differences by time and group treatment. P-value below 0.05 were considered as statistical differences between groups.

**Figure 2:**
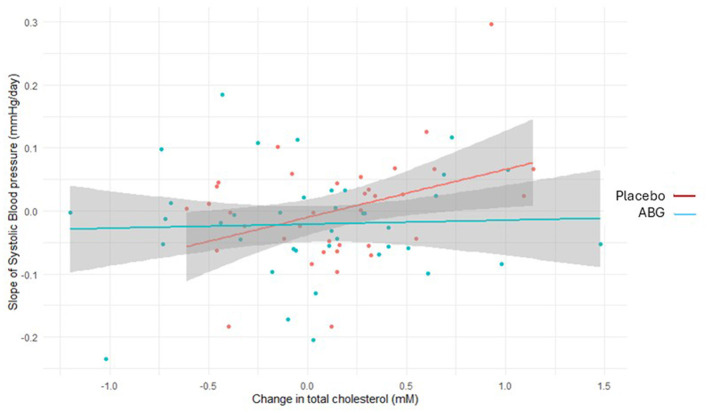
Association between change in total cholesterol and the slope of systolic blood pressure according to study group. Each point represents an individual participant. The x-axis shows the change in total cholesterol (mM), and the y-axis shows the slope of systolic blood pressure (mmHg/day). Linear regression lines (with 95% confidence bands) are shown separately for the placebo group and the ABG group. Slope estimates derive from a multiple linear regression model adjusted for prior lipid-lowering medication (overall model *R*^2^ = 0.103, adjusted *R*^2^ = 0.052; *F*_(4, 70)_ = 2.02, *p* = 0.102). Placebo group slope = 0.080 (95% CI: 0.015 to 0.145); Δcholesterol × group interaction term (difference in slopes) = −0.075 (95% CI: −0.155 to 0.006; *p* = 0.0703). The interaction did not reach the pre-specified significance threshold (*p* < 0.05) and should be regarded as exploratory and unconfirmed.

A multiple linear regression model with a Δcholesterol × group interaction term was fitted to evaluate the association between the change in blood pressure and the change in lipoproteins, adjusting for prior lipid-lowering medication. The overall model showed a modest and statistically non-significant fit [*R*^2^ = 0.103, adjusted *R*^2^ = 0.052; *F*_(4, 70)_ = 2.02, *p* = 0.102]. The change in systolic blood pressure was directly correlated with the change in blood total cholesterol levels (*p* = 0.0166) and LDL-C (*p* = 0.0495). The Δcholesterol × group interaction term did not reach the pre-specified significance threshold of *p* < 0.05 (*p* = 0.0703; 95% CI for the difference in slopes: −0.155 to 0.006) and should therefore not be interpreted as a confirmed differential association between cholesterol and blood pressure across groups; this exploratory observation is illustrated in Figure 2. Similar analysis were performed with other lipoprotein subclasses, however, none of them showed statistical significance (for example with systolic blood pressure HDL-C (*p* = 0.0969); TG (*p* = 0.977); and diastolic blood pressure Total-cholesterol (*p* = 0.058); LDL-C (*p* = 0.162); HDL-C (*p* = 0.122) and TG (*p* = 0.969).

Endothelial function parameters (RHI and AI75) were measured at baseline and after 12 weeks of intervention. Neither parameter showed a statistically significant within-group change or a significant group × time interaction. RHI changed from 2.059 ± 0.414 to 2.033 ± 0.597 in the placebo group (*p* = 0.9583) and from 2.016 ± 0.569 to 2.068 ± 0.728 in the ABG group (*p* = 0.8276); the group × time interaction was not significant (*p* = 0.9753). AI_75_ changed from 30.4 ± 14.9 to 26.7 ± 17.2 in the placebo group (*p* = 0.1767) and from 25.0 ± 17.0 to 21.1 ± 15.1 in the ABG group (*p* = 0.1139); the group × time interaction was not significant (*p* = 0.1185). These results indicate that 12 weeks of ABG supplementation did not significantly modify endothelial function, as assessed by EndoPAT™, relative to placebo.

### 3.4 Cluster analysis

**Figure 3:**
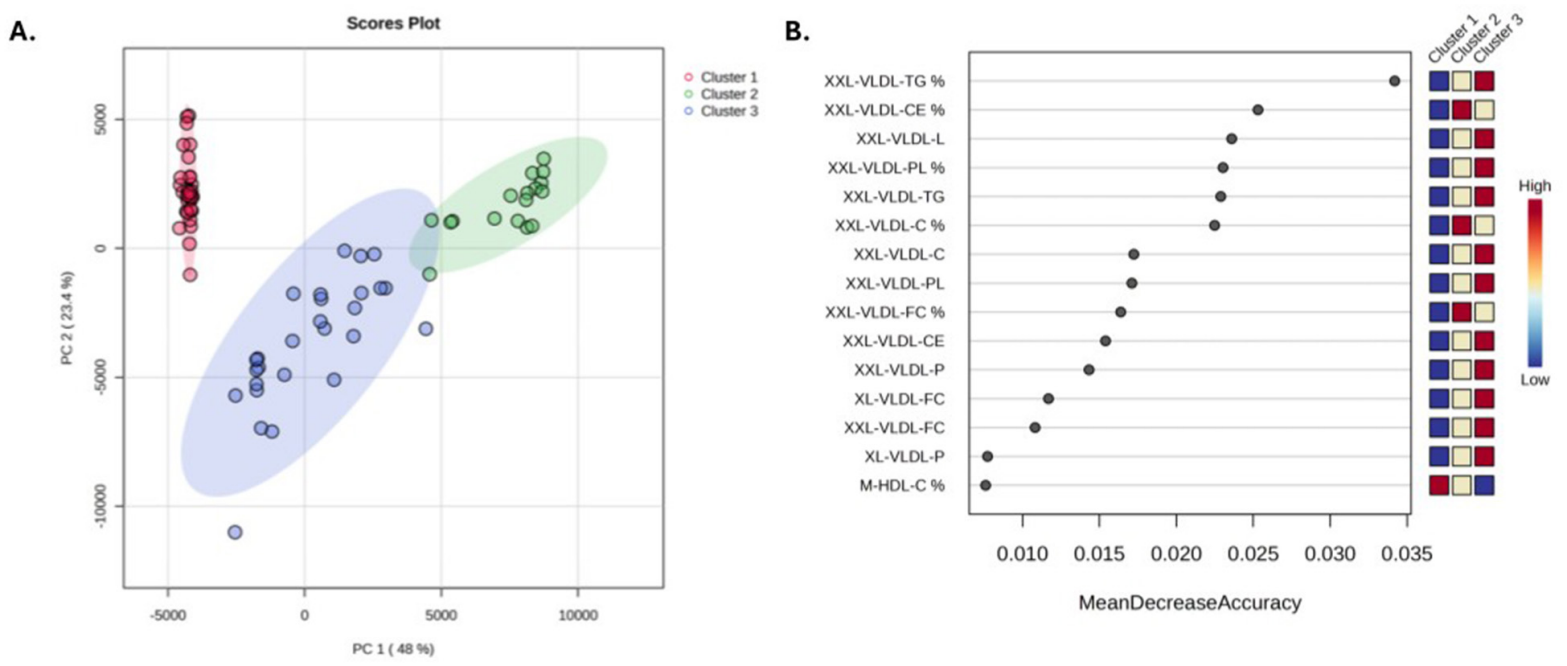
Main lipoprotein subclasses that identify each cluster. **(A)** 2D Score plot of the PLS-DA cluster classification. PLS-DA cross validation for 5 components: Accuracy 0.9875; R2 0.9692; and Q2 0.9590. **(B)** Variable importance in projection (VIP) and weighted sum of absolute regression coefficients. The colored boxes on the right indicate the relative concentrations of the corresponding metabolite in each group under study.

Considering that the response to treatment may be influenced by the baseline lipoprotein levels, a cluster analysis was conducted to classify the volunteers based on their lipoprotein profile. Three distinct clusters were identified, each with differential characteristics of metabolic syndrome, as shown in Table 5. For example, Cluster 1 shows a lower incidence of metabolic syndrome and is characterized by lower levels of body mass index, blood lipids, LP-IR score and augmentation index (AI_75_). On the opposite end, Cluster 3 is characterized by having more than three components of the metabolic syndrome classification according to NCEP ATP III, which are higher body mass index, high blood glucose, low HDL-cholesterol and high blood triacylglycerides levels, LP-IR score and glycoprotein acetyls a biomarker of inflammation. Main differences in lipoprotein subclasses are described in Figure 3. The PLS-DA confirmed that each cluster could be identified by a specific lipid profile, being the main variables in importance that may classify each cluster the XXL-VLDL lipoprotein. In which, Cluster 3 is characterized by XXL-VLDL lipoproteins with high levels of TG, lipids and phospholipids; while, on the other hand, Cluster 1 present lower levels.

**Table 5:** Clusters demographic characteristics at base line.

Variable	Cluster 1 (*n* = 31)	Cluster 2 (*n* = 17)	Cluster 3 (*n* = 27)	*p-value*
Metabolic syndrome	8 (26%)	6 (35%)	16 (60%)	0.0328
Treatment (Placebo:ABG)	18:13	7:10	11:16	0.3698
Age (years)	63.6 ± 5.7	65.5 ± 4.9	62.9 ± 6.2	0.3236
Sex (male:female)	16:15	6:11	18:9	0.1369
Systolic blood pressure (mmHg)	145.2 ± 18.9	144.8 ± 20.3	151.2 ± 22.2	0.4671
Diastolic blood pressure (mmHg)	85.7 ± 11.9	86.0 ± 9.7	85.1 ± 11.1	0.9656
Body mass index (kg/m^2^)	29.5 ± 4.5	30.3 ± 4.1	34.9 ± 4.8^a****, b**^	< 0.0001
RHI	2.06 ± 0.54	1.94 ± 0.36	2.08 ± 0.51	0.6264
AI_75_	31.7 ± 13.4	31.2 ± 15.8	20.7 ± 17.2^a*^	0.0196
Glycoprotein Acetyls (mM)	0.85 ± 0.09	0.88 ± 0.06	0.96 ± 0.12^a***, b*^	0.0002
Blood glucose (mg/dL)	107.6 ± 14.5	100.3 ± 8.2	111.7 ± 16.5^b*^	0.0426
Blood cholesterol (mM)	4.77 ± 0.88	5.86 ± 1.12^a***^	5.10 ± 0.84^b*^	0.0010
HDL-cholesterol (mM)	1.59 ± 0.43	1.47 ± 0.28	1.20 ± 0.24^a***, b*^	0.0002
LDL-cholesterol (mM)	1.91 ± 0.42	2.52 ± 0.56^a***^	2.21 ± 0.44^a*^	0.0002
Blood triacylglycerides (mM)	0.88 ± 0.23	1.38 ± 0.26^a**^	1.95 ± 0.70^a****, b***^	< 0.0001
LP-IR score	36.4 ± 4.3	40.6 ± 2.5^a***^	43.2 ± 2.0^a****, b*^	< 0.0001

Data is presented as mean ± standard deviation. Differences between clusters for continuous variables was determined by one-way ANOVA. Bonferroni's multiple comparison test was used to determine differences by group. For categorical variables a Chi-squared test was performed. P-value below 0.05 were considered as statistical differences between groups. Differences between clusters were denoted with superscript as follows: ^a^denote difference with Cluster 1, and ^b^denote difference with Cluster 2; while ^*^, ^**^, ^***^, and ^****^ denotes p-values of < 0.05, < 0.01, < 0.001, and < 0.0001, respectively.

It should be noted that Cluster 2 comprised only 17 participants (ABG: *n* = 10; Placebo: *n* = 7), a sample size that substantially limits statistical power; results for this subgroup in Table 6 should therefore be interpreted with caution. The within-cluster treatment comparisons reported below represent *post-hoc* subgroup analyses within an already exploratory clustering framework; no correction for multiple comparisons was applied, and *p-values* should therefore be interpreted as hypothesis-generating rather than confirmatory. With this caveat in mind, a significant reduction in triglyceride levels was observed in Cluster 3 treated with ABG (*p*= 0.0154), with a significant difference between clusters in the ABG treatment group (*p* = 0.0316). However, it is also observed that volunteers in Cluster 1 treated with ABG show a significant increase in glucose levels (Table 6). Considering that the main effect of ABG treatment was a decrease in triglycerides, an analysis of the lipoprotein profile in VLDL lipoproteins was performed. It was observed that in Cluster 3, which is characterized by high levels of XXL-VLDL, ABG treatment reduces the lipid content in these particles (*p* = 0.0155) as well as the TG of VLDL particles (*p* = 0.0131). Additionally, a significant increase in lipid content (*p* = < 0.0001), mainly phospholipids (*p* = 0.0388), in XL-HDL particles were observed.

**Table 6:** Changes in metabolic syndrome parameters derived by treatment and cluster belonging.

Variable	Cluster 1			Cluster 2			Cluster 3			*p-value* for cluster
	Pre-treatment	Post-treatment	*p-value*	Pre-treatment	Post-treatment	*p-value*	Pre-treatment	Post-treatment	*p-value*	
**Group Placebo**	*n* = 18			*n* = 7			*n* = 11			
Systolic blood pressure (mmHg)	144 ± 16	140 ± 13	0.3054	144 ± 12	139 ± 12	0.4246	149 ± 22	140 ± 14	0.0523	0.6602
Diastolic blood pressure (mmHg)	85 ± 12	85 ± 11	0.7799	84 ± 3	83 ± 10	0.8227	85 ± 13	82 ± 9	0.3080	0.8117
Glycoprotein acetyls (mM)	0.87 ± 0.09	0.85 ± 0.08	0.6981	0.86 ± 0.06	0.90 ± 0.07	0.5081	0.98 ± 0.14	0.98 ± 0.12	0.9793	0.7440
Blood glucose (mg/dL)	109 ± 15	103 ± 15	0.0058	102 ± 10	103 ± 8	0.6548	107 ± 11	110 ± 17	0.8026	0.0626
Blood cholesterol (mM)	4.62 ± 0.75	4.76 ± 0.87	0.1872	5.49 ± 1.17	5.59 ± 1.12	0.5588	5.09 ± 1.09	5.24 ± 1.04	0.2581	0.9663
HDL-cholesterol (mM)	1.53 ± 0.33	1.57 ± 0.38	0.2526	1.39 ± 0.39	1.38 ± 0.36	0.8867	1.20 ± 0.32	1.23 ± 0.34	0.4153	0.7508
LDL-cholesterol (mM)	1.87 ± 0.37	1.92 ± 0.37	0.3905	2.35 ± 0.43	2.39 ± 0.46	0.6636	2.22 ± 0.57	2.31 ± 0.51	0.1941	0.8483
Triacylglycerides (mM)	0.86 ± 0.25	0.88 ± 0.18	0.7824	1.31 ± 0.24	1.42 ± 0.29	0.2848	2.00 ± 0.73	1.95 ± 0.93	0.4847	0.4413
LP-IR score	36.7 ± 3.9	36.8 ± 3.8	0.6610	41.3 ± 2.1	41.4 ± 1.6	0.8145	43.5 ± 2.6	42.5 ± 3.0	0.0680	0.1965
**Group ABG**	*n* = 13			*n* = 10			*n* = 16			
Systolic blood pressure (mmHg)	147 ± 23	142 ± 17	0.1500	147 ± 25	139 ± 18	0.1085	153 ± 23	147 ± 20	0.0885	0.9754
Diastolic blood pressure (mmHg)	86 ± 12	84 ± 7	0.3636	87 ± 12	84 ± 8	0.1802	85 ± 10	83 ± 10	0.3970	0.8908
Glycoprotein acetyls (mM)	0.82 ± 0.09	0.86 ± 0.11	0.4018	0.89 ± 0.06	0.92 ± 0.10	0.4409	0.94 ± 0.10	0.92 ± 0.08	0.4177	0.4015
Blood glucose (mg/dL)	105 ± 15	109 ± 19	0.3279	99 ± 7	103 ± 25	0.3657	115 ± 19	110 ± 20	0.2569	0.2331
Blood cholesterol (mM)	4.99 ± 1.02	5.06 ± 1.12	0.6600	6.12 ± 1.07	6.10 ± 0.97	0.9269	5.11 ± 0.66	5.17 ± 0.60	0.6698	0.9255
HDL-cholesterol (mM)	1.67 ± 0.56	1.65 ± 0.65	0.5208	1.54 ± 0.17	1.54 ± 0.21	0.9808	1.21 ± 0.16	1.26 ± 0.18	0.1248	0.3021
LDL-cholesterol (mM)	1.98 ± 0.48	2.01 ± 0.50	0.5208	2.64 ± 0.60	2.63 ± 0.58	0.8923	2.22 ± 0.34	2.24 ± 0.31	0.7670	0.9481
Triacylglycerides (mM)	0.91 ± 0.22	1.02 ± 0.23	0.3108	1.44 ± 0.28	1.53 ± 0.55	0.4697	1.92 ± 0.70	1.67 ± 0.52	0.0154	0.0316
LP-IR score	36.0 ± 4.9	36.5 ± 8.3	0.5986	40.0 ± 2.8	39.7 ± 4.7	0.7967	43.0 ± 1.5	42.4 ± 2.1	0.4984	0.6906
Systolic blood pressure (mmHg)	−2.5	−14.5 to 9.5	0.6804	−2.0	−18.3 to 14.3	0.8037	10.7	−2.2 to 23.7	0.1027	0.6084
Diastolic blood pressure (mmHg)	−1.0	−7.6 to 5.6	0.7727	−2.5	−11.5 to 6.4	0.5750	3.8	−3.3 to 10.9	0.2847	0.9572
Glycoprotein acetyls (mM)	0.04	−0.01 to 0.10	0.1002	−0.003	−0.07 to 0.07	0.9418	−0.028	−0.08 to 0.03	0.3241	0.1753
Blood glucose (mg/dL)	10.2	0.9 to 19.5	0.0313	−3.7	−16.2 to 8.7	0.5478	1.1	−8.6 to 11.0	0.8278	0.4150
Blood cholesterol (mM)	−0.07	−0.45 to 0.30	0.7110	−0.12	−0.63 to 0.40	0.6506	−0.09	−0.50 to 0.31	0.6508	0.4622
HDL-cholesterol (mM)	−0.06	−0.15 to 0.03	0.2157	0.01	−0.12 to 0.14	0.9000	0.02	−0.08 to 0.12	0.7192	0.7308
LDL-cholesterol (mM)	−0.02	−0.22 to 0.17	0.7984	−0.05	−0.32 to 0.22	0.6962	−0.07	−0.29 to 0.14	0.4940	0.4472
Triacylglycerides (mM)	0.09	−0.15 to 0.34	0.4388	−0.01	−0.34 to 0.31	0.9261	−0.19	−0.45 to 0.06	0.1427	0.6384
LP-IR score	0.37	−2.57 to 3.3	0.7976	−0.44	−2.45 to 1.57	0.6455	0.28	−0.95 to 1.52	0.6400	0.8888

Data is presented as mean ± standard deviation or mean difference of the change of each parameter (ABG – Placebo) and 95% confidence interval of the difference. Differences between treatments and clusters was determined by two-way ANOVA. Bonferroni's multiple comparison test was used to determine differences by treatment/cluster. P-values below 0.05 was considered as statistical difference.

## 4 Discussion

The present study evaluated the effect of supplementation with an optimized ABG extract on conventional lipid markers and the detailed lipoprotein subclass profile in subjects with Grade I hypertension. With respect to the primary outcome, no statistically significant changes were observed in any conventional lipid marker after 12 weeks of ABG supplementation (Table 3), a finding consistent with the three previously published clinical trials using ABG at various doses (30–32). This null result at the conventional lipid level is informative and should not be interpreted as an absence of biological activity; rather, it underscores that standard lipid panels may lack the resolution to detect subtle lipoprotein compositional changes that characterize the early effects of functional food interventions at low doses. The secondary exploratory analyses of NMR-derived lipoprotein subclasses, discussed below, provide mechanistic context for this observation. Since 1993, a total of 16 meta-analyses has been conducted on the effect of garlic consumption on lipid profiles. The results have varied, with some studies demonstrating significant reductions in cholesterol levels (33), while others have observed no effect (34). The main limitations of these meta-analyses are the lack of information regarding the type of garlic used, as well as the standardization of its content and bioactive compounds. Most studies suggest that effectiveness may be limited to a specific population group, particularly older adults and individuals with hyperlipidemia (22). Likewise, effective doses are estimated to range between 500 and 1,000 mg of garlic per day, doses that are not well tolerated by many individuals.

The importance of this study lies in the fact that it provides evidence on ABG in the lipid profile, for which there is currently limited information (only three clinical trials). Two of these trials use high doses of ABG (6 and 12 g per day) (30, 31), while one employs the same dose used in this study (32). In all three studies mentioned, no significant changes in the blood lipid profile were observed. The present study provides relevant information on an ABG extract with a low and standardized dose of S-allylcysteine (0.25 mg/day) which was highly tolerated by study participants, as well as the observation of its effects on the blood lipoprotein profile subclasses. To this regard, detailed analyses of lipoprotein subclasses showed a reduction in total lipoprotein particles, which may reflect a lower number of atherogenic particles and is consistent with a reduced cardiovascular risk (35). Although HDL particle numbers also decreased, this was accompanied by compositional changes—higher phospholipid and lower triglyceride content in large HDL—directionally consistent with more mature HDL particles (36). In addition, the compositional remodeling observed in XXL-VLDL particles—specifically, a mean reduction of 13.8 percentage points in total cholesterol percentage (95% CI: 9.6–18.1) and 13.1 percentage points in esterified cholesterol percentage (95% CI: 8.8–17.3), with a concomitant increase of 11.3 percentage points in triglyceride percentage (95% CI: 7.1–15.5; all *p* < 0.0001, Table 4)—is consistent with cholesterol redistribution from the largest VLDL particles, a pattern mechanistically plausible given the known roles of CETP and hepatic lipase in lipoprotein remodeling (37) The complementary changes in large HDL particles—a mean increase of 2.0 percentage points in phospholipid content (95% CI: 0.2–3.7; *p* = 0.0277) and a reduction of 2.6 percentage points in triglyceride content (95% CI: 0.9–4.4; *p* = 0.0036)—are directionally consistent with more mature HDL particles, a pattern directionally consistent with enhanced reverse cholesterol transport capacity (38). Overall, these compositional shifts are directionally consistent with lipoprotein remodeling patterns associated with reduced atherogenic risk in the epidemiological literature. However, the clinical significance of changes of this magnitude has not been established in prospective outcomes trials, and these findings should not be interpreted as evidence of cardiovascular benefit. They are best regarded as mechanistic signals warranting investigation in future adequately powered studies with pre-specified NMR-based endpoints and hard cardiovascular outcomes. It is also important to note that HDL functionality (e.g., cholesterol efflux capacity or ApoA1 functional activity) was not directly assessed in this study; the compositional changes observed should not be equated with improved HDL function in the absence of direct functional assays.

It is important to acknowledge that 0.25 mg/day of SAC is lower than doses employed in some prior clinical studies on garlic extracts; however, this dose should be interpreted in the context of the pharmacokinetic properties of SAC and the multi-compound nature of ABG. SAC is well absorbed following oral administration, exhibiting high bioavailability, rapid systemic distribution, and urinary excretion as the primary elimination route across a wide dose range (39). Critically, the biological effects of ABG cannot be attributed solely to SAC at this dose. The aging process generates a complex bioactive matrix—including S-1-propenylcysteine, S-allylmercaptocysteine, polyphenols, flavonoids, and melanoidins—whose collective antioxidant, vasodilatory, and ACE-inhibitory properties are well documented (40), and likely contribute synergistically to the observed effects at a dose that would be considered sub-therapeutic for SAC alone. Standardizing by SAC content ensures batch-to-batch reproducibility and comparability with the existing literature without implying it is the sole active constituent, consistent with established practice in nutraceutical research.

Consistent with prior evidence, the present study shows that longitudinal changes in cholesterol levels track closely with concomitant changes in blood pressure (41, 42). Against this background, the exploratory observation that ABG treatment attenuated the positive coupling between temporal trajectories of cholesterol and systolic blood pressure (Figure 2; interaction *p* = 0.0703, not statistically significant) is consistent with the hypothesis that this intervention could plausibly influence shared pathophysiological pathways linking dyslipidaemia to vascular dysfunction and hypertension. However, this remains speculative and should be confirmed in adequately powered studies.

It was observed that ABG treatment attenuated the positive coupling between temporal trajectories of cholesterol and systolic blood pressure suggests that this intervention may disrupt shared pathophysiological pathways linking dyslipidaemia to vascular dysfunction and hypertension.

Similarly to previous studies on the impact of ABG on lipid profiles, our findings indicate that the observed modifications in lipoprotein composition may depend on the individual's baseline metabolic status. The cluster analysis performed identified that subgroups of patients with different metabolic characteristics presented varied responses to the intervention. Notably, participants classified in Cluster 3, characterized by a more adverse metabolic profile, showed a significant reduction in triglyceride levels after ABG supplementation. This finding is relevant since elevated triglyceride levels in very-low-density lipoprotein (VLDL) fractions are associated with increased cardiovascular risk (43). These findings highlight the importance of considering metabolic heterogeneity in the design of future investigations on the effects of black garlic on cardiovascular health.

A secondary finding that warrants dedicated discussion is the nominally significant increase in fasting blood glucose observed in Cluster 1 participants receiving ABG (+10.2 mg/dL; 95% CI: 0.9–19.5; *p* = 0.0313). This observation requires careful contextualization. First, across the full study sample, ABG supplementation was not associated with a statistically significant change in blood glucose compared to placebo (mean change: ABG +1.1 ± 14.9 mg/dL vs. Placebo −2.9 ± 8.5 mg/dL; *p* = 0.17), indicating no global hyperglycaemic signal. Second, baseline glucose levels in both groups were already in the impaired fasting glucose range (ABG: 108.0 ± 16.4 mg/dL; Placebo: 106.0 ± 12.5 mg/dL), which is consistent with the cardiometabolic profile of adults with Grade I hypertension and increases the inherent variability of glucose measurements. Third, to our knowledge, no published study has reported a hyperglycaemic effect of aged black garlic or its principal bioactive compound S-allylcysteine (SAC). On the contrary, preclinical and clinical evidence consistently attributes mild hypoglycaemic properties to garlic-derived compounds (23), potentially mediated through inhibition of α-glucosidase, enhancement of insulin sensitivity, or antioxidant protection of pancreatic β-cells (44). The observation of a glucose increase specifically in the metabolically healthiest subgroup (Cluster 1), and its absence at the whole-sample level, is therefore biologically counterintuitive and not supported by any known mechanism of action of ABG. Taken together, we interpret this finding as most likely representing a statistical artifact attributable to the small subgroup size, the absence of multiple comparisons correction, and inherent fasting glucose variability, rather than a genuine biological effect. This finding should be monitored in future adequately powered studies.

This study has several limitations that we consider should be contemplated for further studies with the aim to validate the lipid lowering effect of ABG extracts. First, it seems that the main effects may be observed in patients with impaired lipid control and metabolism. Therefore, further studies should include as the main inclusion criteria subjects with hypercholesterolemia and more than three components of the metabolic syndrome classification according to the NCEP ATPIII. Regarding the inclusion of volunteers with lipid-lowering therapy, our data suggests that no differences in treatment were observed due to drug therapy. However, safety consideration should be taken since garlic may improve statin half-life and side-effects. Second, the cluster-based subgroup analyses reported here are *post-hoc* in nature and were conducted within an already exploratory unsupervised clustering framework. No correction for multiple comparisons was applied to the within-cluster treatment comparisons; consequently*, p-values* should be regarded as hypothesis-generating and interpreted with caution. Third, Cluster 2 included only 17 participants (ABG: *n* = 10; Placebo: *n* = 7), resulting in substantially limited statistical power for this subgroup; findings for Cluster 2 in Table 6 are therefore exploratory and should not be interpreted as definitive. Future adequately powered studies with pre-specified subgroup hypotheses are needed to confirm the differential treatment response across metabolic clusters. Fourth, although NMR-derived lipoprotein variables were organized into 14 biologically coherent families for analysis, no formal correction for multiple comparisons across families was applied. The probability of false-positive findings across the 14 ANOVAs performed cannot be fully excluded, and all findings from the lipoprotein profiling analysis should therefore be interpreted as exploratory and hypothesis-generating, pending replication in an independent, adequately powered cohort. Fifth, no independent data monitoring committee was established for this study; the absence of external oversight is acknowledged as a limitation in the context of industry-sponsored research, and findings should be interpreted accordingly. Sixth, allocation followed a systematic, sex-stratified sequential procedure rather than computer-generated block randomization, and no formal allocation concealment mechanism was implemented; while the triple-blind design was maintained throughout data collection and analysis, these aspects represent methodological limitations relative to CONSORT-recommended practice. Seventh, statistical analyses were performed on a per-protocol basis (*n* = 75 of 89 randomized participants); an intention-to-treat analysis was not conducted. Although attrition did not differ significantly between treatment arms (13.3% in ABG vs. 18.2% in placebo; *p* = 0.57), the absence of an ITT analysis may affect the conservativeness and generalizability of the effect estimates reported. Eighth, the exclusion of patients receiving ACE inhibitors and ARBs—which represent the most commonly prescribed antihypertensive drug classes—limits the generalizability of our findings to patients with Grade I hypertension managed without RAS-inhibiting agents. Future studies should prospectively evaluate the safety and efficacy of ABG supplementation in this broader and clinically representative population (45, 46).

## 5 Conclusions

Twelve weeks of supplementation with a low-dose optimized ABG extract did not produce statistically significant changes in conventional lipid markers (total cholesterol, LDL-C, HDL-C, or triglycerides) in patients with Grade I hypertension, which constituted the primary outcome of this study. In secondary exploratory analyses, ABG supplementation was associated with nominally significant compositional remodeling of lipoprotein subclasses, including a reduction in the cholesterol load of large VLDL particles and an increase in HDL phospholipid content. Exploratory cluster analyses further suggest that the treatment response may be modulated by participants' baseline metabolic profile, with individuals presenting a more adverse lipid phenotype showing greater reductions in triglyceride-rich lipoprotein parameters. These secondary and exploratory findings are hypothesis-generating and should not be interpreted as evidence of clinical efficacy. Adequately powered trials with pre-specified NMR-based endpoints are warranted to confirm whether ABG may offer cardiometabolic benefits in specific high-risk subgroups.

## Data Availability

The raw data supporting the conclusions of this article will be made available by the authors, without undue reservation.
